# Relationships of omega-3 and omega-6 polyunsaturated fatty acids with esophageal diseases: a two-sample Mendelian randomization analysis

**DOI:** 10.3389/fnut.2024.1408647

**Published:** 2024-07-17

**Authors:** Weiming Chen, Maohui Chen, Jin Huang, Qichang Xie, Yizhou Huang, Chun Chen, Yong Zhu

**Affiliations:** ^1^Department of Thoracic Surgery, Fujian Medical University Union Hospital, Fuzhou, China; ^2^Key Laboratory of Cardio-Thoracic Surgery (Fujian Medical University), Fujian Province University, Fuzhou, China; ^3^National Key Clinical Specialty of Thoracic Surgery, Fuzhou, China; ^4^Department of Thoracic Surgery Nursing, Fujian Medical University Union Hospital, Fuzhou, China

**Keywords:** omega-3, omega-6, docosahexaenoic acid, esophageal cancer, esophageal disease, Mendelian randomization

## Abstract

**Introduction:**

Omega-3 polyunsaturated fatty acids (PUFAs) have been widely studied and used as nutritional supplements because of their anti-inflammatory effects. Previous studies have shown an association between polyunsaturated fatty acids such as omega-3 and omega-6 PUFAs with the development of malignant tumors. However, the relationships of omega-3 and omega-6 PUFAs with esophageal diseases have not been characterized.

**Methods:**

Mendelian randomization (MR) is a statistical method for identifying instrumental variables (IVs) from genome-wide association study (GWAS) data, and is associated with little confounding by environmental or other disease-related factors. We used genome-wide association study (GWAS) data from previously published studies on circulating concentrations of omega-3, omega-6, docosahexaenoic acid (DHA) and linoleic acid (LA), as well as esophageal cancer and other esophageal diseases, which were downloaded from the IEU OpenGwas database (https://gwas.mrcieu.ac.uk/) and the GWAS Catalog database (https://www.ebi.ac.uk/). The inverse variance-weighted approach was used as the principal analysis, and the MR–Egger and weighted median methods were used alongside. A series of sensitivity analyses were used to ensure the robustness of the causality estimates.

**Results:**

We found that the circulating omega-3 PUFAs concentration was positively associated with esophageal cancer (*p* = 8 × 10^−4^), and circulating DHA concentration (the main component of omega-3 in food), was also positively associated with esophageal cancer (*p* = 2 × 10^−2^), but no significant association was found between circulating omega-6 PUFAs and esophageal cancer (*p* = 0.17), and circulating LA concentration (the main component of omega-6 in food), was also no significant associated with esophageal cancer (*p* = 0.32). We found no significant relationships of circulating omega-3 and omega-6 PUFAs concentration with four other esophageal diseases.

**Conclusion:**

This study indicates that higher levels of circulating omega-3 PUFAs and DHA concentrations may be a risk factor for the development of esophageal cancer. Conversely, an increased omega-6/omega-3 ratio may serve as a protective factor against esophageal cancer. These findings have significant implications for the clinical application of omega-3 PUFAs and the prevention and treatment of esophageal cancer.

## 1 Introduction

Esophageal cancer is a global health problem, and in a global study of cancer incidence trends, the top three 5 year survival rates for esophageal cancer were in Japan (36%), China (34%), and South Korea (31%), while all other countries had rates of <30% (1). Esophageal cancer can be associated with Barrett’s esophagus, a history of gastroesophageal reflux disease, obesity, smoking, and alcohol consumption; and its incidence and mortality show substantial regional variations (2–4). Esophageal cancer is a type of gastrointestinal tumor, and the ingestion of particular foods is thought to be represent a risk factor for esophageal cancer; for example, betel quid chewing and low intake of fresh fruit and vegetables. Furthermore, the lack of certain micronutrients and long-term dietary habits may also represent risk factors (4, 5). Omega-3 and omega-6 polyunsaturated fatty acids (PUFAs) are commonly used as dietary supplements and have been demonstrated to exhibit significant prophylactic effects against coronary heart disease and asthma (6, 7). Because of their anti-inflammatory properties, omega-3 PUFAs reduce the risk of inflammatory bowel disease (8); and omega-6 PUFAs may reduce the risks of osteoarthritis of the knee and hip, and therefore may have potential for the prevention and treatment of autoimmune diseases (9). However, their effects on the risks of tumors are controversial. Previous studies have shown that PUFAs such as omega-3 and omega-6 PUFAs may increase or reduce the risks of developing specific tumors. They have been shown to increase the risks of prostate and endometrial cancers (10, 11), but to protect against the development of liver, breast, ovarian, and brain tumors (12). However, there have been no studies of the relationship of esophageal cancer with omega-3 and omega-6 PUFAs. Mendelian randomization (MR) is an emerging research methodology that is used to determine whether an association exists between particular exposures and outcomes, and it permits the avoidance of the limitations of residual confounding and reverse causation. SNPs are used as instrumental variables to infer whether a relationship exists between exposures and outcomes. The genetic composition of an individual is determined before birth and is therefore not subject to confounding, and Mendelian randomization (MR) studies rely on the fact that genetic variants are randomly assigned during meiosis, such that an unbiased assessment of exposure-outcome relationships can be made (13). Here, we aimed to characterize the relationships of circulating concentrations of omega-3 and omega-6 PUFA with the development of esophageal cancer.

## 2 Materials and methods

### 2.1 Study design

**Figure 1:**
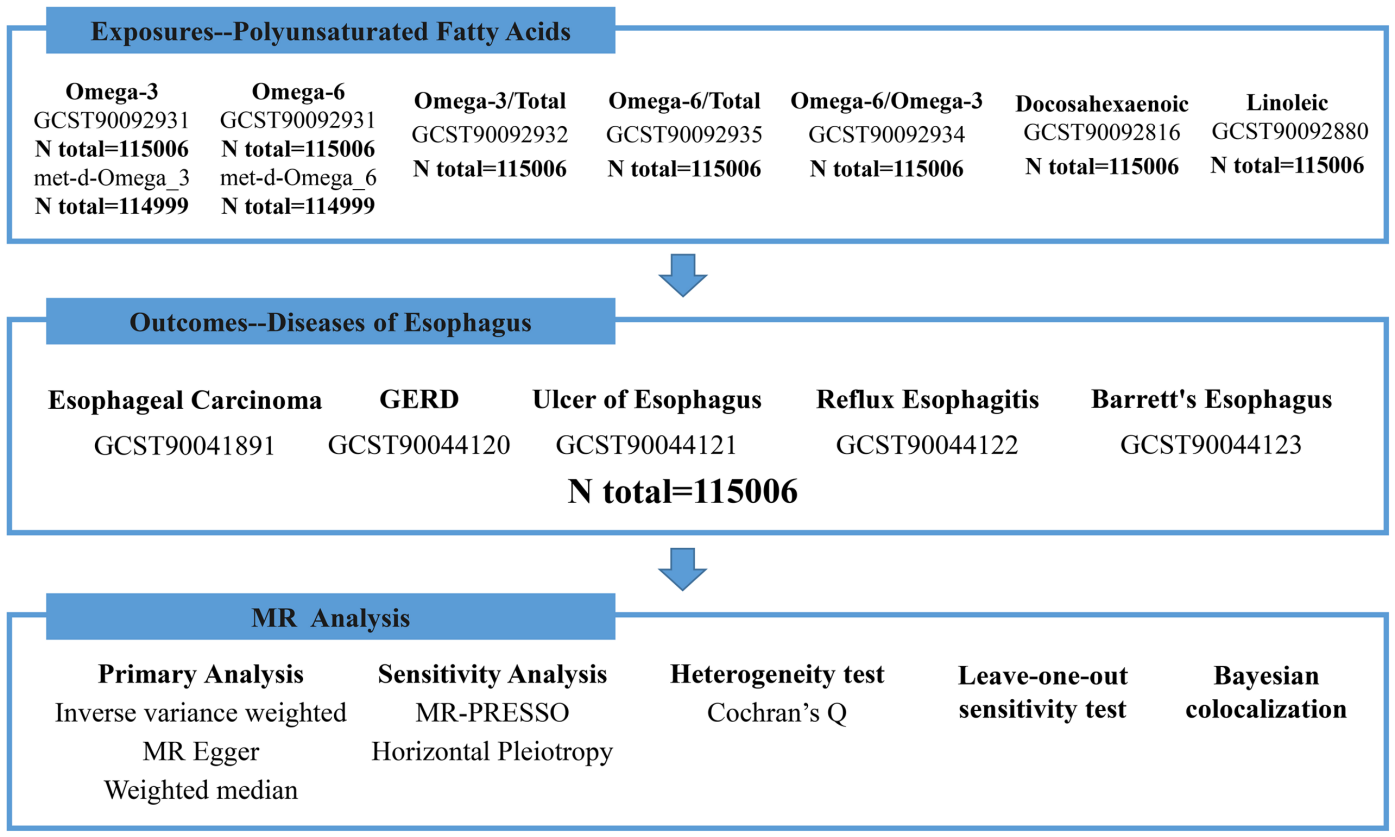
The flow diagram of MR analysis.

We conducted a two-sample MR study of GWAS data obtained from previously published studies on circulating concentrations of omega-3, omega-6, DHA, LA, as well as esophageal cancer and other esophageal diseases which were downloaded from the IEU OpenGwas database^1^ and the GWAS catalog databases.^2^ Therefore, the study did not require approval by the institutional ethics committee. To ensure the robustness of the MR data, we made the following three assumptions: (1) genetic variation is associated with specific exposure factors, (2) genetic variation is not associated with confounding factors, and (3) genetic variation affects the outcomes only through specific risk factors. Figure 1 shows the details of the study design.

### 2.2 Data sources

Data regarding the exposures and outcomes were obtained from the IEU OpenGwas database and the GWAS catalog database. The exposure factors were omega-3 PUFAs (ebi-a-GCST90092931), omega-6 PUFAs (ebi-a-GCST90092933), the omega-3/total fatty acid ratio (ebi-a-GCST90092932), the omega-6/total fatty acid ratio (ebi-a-GCST90092935), and the omega-6/omega-3 ratio (ebi-a-GCST90092934), and the data consisted of 115,006 samples and 11,590,399 SNPs (14). The data regarding omega-3 (met-d-Omega_3) and omega-6 (met-d-Omega_6) PUFAs consisted of 114,999 samples and 12,321,875 SNPs (15). The data regarding DHA (ebi-a-GCST90092816) and linoleic acid levels (ebi-a-GCST90092880) consisted of 115,006 samples and 11,590,399 SNPs (14). The outcome factors comprised esophageal cancer (ebi-a-GCST90041891), gastroesophageal reflux disease (ebi-a-GCST90044120), ulcer of the esophagus (ebi-a-GCST90044121), reflux esophagitis (ebi-a-GCST90044122), and Barrett’s esophagus(ebi-a-GCST90044123), and the data consisted of 456,348 samples and 11,842,647 SNPs related to these (16). Further information regarding the exposure and outcome factors are presented in Tables 1, 2, respectively.

**Table 1:** Characters of esophageal disease.

Disease	Study	Journal	Sample	SNPs	GWAS ID
Esophageal carcinoma	Jiang et al.	Nat Genet	456,276	11,842,647	ebi-a-GCST90041891
GERD	Jiang et al.	Nat Genet	456,348	11,842,647	ebi-a-GCST90044120
Ulcer of esophagus	Jiang et al.	Nat Genet	456,348	11,842,647	ebi-a-GCST90044121
Reflux esophagitis	Jiang et al.	Nat Genet	456,348	11,842,647	ebi-a-GCST90044122
Barrett’s esophagus	Jiang et al.	Nat Genet	456,348	11,842,647	ebi-a-GCST90044123

**Table 2:** Characters of polyunsaturated fatty acid (PUFA).

PUFA	Study	Journal	Sample	SNPs	GWAS ID
Omega-3	Richardson et al.	PLoS Biol	115,006	11,590,399	ebi-a-GCST90092931
Omega-3	Borges et al.	Web	114,999	12,321,875	met-d-Omega_3
Omega-6	Richardson et al.	PLoS Biol	115,006	11,590,399	ebi-a-GCST90092933
Omega-6	Borges et al.	Web	114,999	12,321,875	met-d-Omega_6
Docosahexaenoic	Richardson et al.	PLoS Biol	115,006	11,590,399	ebi-a-GCST90092816
Linoleic	Richardson et al	PLoS Biol	115,006	11,590,399	ebi-a-GCST90092880
Omega-3/Total	Richardson et al.	PLoS Biol	115,006	11,590,399	ebi-a-GCST90092932
Omega-6/Total	Richardson et al.	PLoS Biol	115,006	11,590,399	ebi-a-GCST90092935
Omega-6/Omega-3	Richardson et al.	PLoS Biol	115,006	11,590,399	ebi-a-GCST90092934

### 2.3 Selection of instrumental variables

We rigorously selected genetic variants that showed close associations with circulating concentrations of omega-3 and omega-6 (genetic correlation: *p* < 5 × 10^−8^) to obtain complete and reliable results. We also further performed quality control using chain disequilibrium (*r*^2^ < 0.001, 10,000 kb) to ensure that single-nucleotide polymorphisms (SNPs) within a specific window were pruned to assess the bias caused by the residual LD of the genetic variants. The *F*-statistic represents the closeness of a correlation, and it is generally considered that SNPs with *F* > 10 are closely associated with the exposure factors. The formula for calculating the *F*-statistic and *R*^2^is as follows (17). The *F*-statistics for all of the SNPs included in the study were calculated and found to be >10 and part of the *F* values were shown in Supplementary Table S1.

$$F-\mathrm{statistic}=\frac{R^{2}\left(N-2\right)}{1-R^{2}}$$

$$R^{2}=\frac{2\times EAF\times \left(1-EAF\right)\times \beta^{2}}{2\times EAF\times \left(1-EAF\right)\times \beta^{2}+2\times EAF\times \left(1-EAF\right)\times N\times {SE}^{2}}$$

### 2.4 MR analysis

To determine whether the association of omega-3 and omega-6 PUFA concentrations with esophageal cancer, we used a number of different methods in the two-sample MR analysis. The inverse variance-weighted (IVW) method was used for the primary analysis; this method involves ignoring the intercept in regression and using the inverse of the variance of the outcome for fitting. Therefore, it may be possible to identify a relationship between an exposure and an outcome despite heterogeneity of the IVs, which may yield biased results. Consequently, we used additional methods for the MR analysis (the MR–Egger and weighted median), to overcome the drawbacks of using IVW alone. The biggest difference between the MR–Egger and IVW methods is that the presence of the intercept is taken into account in the regression in the former, but it also uses the inverse of the variance of the outcome for fitting. The weighted median method is able to provide an unbiased estimate of the effects, and therefore it represents a good complementary method of analysis.

### 2.5 Complementary analysis methods

The central idea of Mendelian randomization is that IVs can only influence the outcome through exposure factors, but if IVs can influence outcomes through an alternative route, there is horizontal multiplicity of results. Therefore, we used MR-PRESSO to conduct a test of pleiotropy, which is also a means of sensitivity testing, as well as two other sensitivity testing methods: the heterogeneity test and the leave-one-out sensitivity test. The heterogeneity test, also known as Cochran’s *Q* test, is used to determine whether there is heterogeneity among the IVs, while the leave-one-out sensitivity test calculates the MR results of the remaining IVs after removing each IV one by one. It is thus used to investigate the effect of individual IVs on the overall effect.

### 2.6 Colocalization analysis

We conducted a colocalization analysis to evaluate whether shared SNPs exist with omega-3 and esophageal cancer at common genomic loci. For each SNP associated with omega-3, we performed a colocalization analysis within a 500 kb range upstream and downstream of the genomic region. Our analysis results conform to the following four hypotheses: H0 (the genomic locus is not associated with either trait), H1 (associated with esophageal cancer but not with omega-3), H2 (associated with omega-3 but not with esophageal cancer), H3 (associated with both omega-3 and esophageal cancer through two different SNPs), and H4 (associated with both omega-3 and esophageal cancer through a shared SNP).

## 3 Results

### 3.1 Association of omega-3 and omega-6 PUFAs with esophageal cancer

We performed two sets of MR analyses of the relationships of omega-3 and omega-6 PUFAs with esophageal cancer. In the first set, we chose not to use proxy SNPs, and finally 48 and 56 SNPs were entered into the MR analyses, respectively. We found a positive correlation between circulating omega-3 PUFA concentration and the risk of esophageal cancer, but no significant correlation was found between the circulating omega-6 concentration and the risk of esophageal cancer. The IVW analyses showed close correlations between omega-3 PUFA concentration and esophageal cancer (OR = 2.16, 95% CI = 1.38–3.38, *p* = 8 × 10^−4^), and between omega-6 PUFA concentration and esophageal cancer (OR = 1.51, 95% CI = 0.84–2.72, *p* = 0.17). MR-PRESSO analysis did not show evidence of horizontal pleiotropy for the analysis of the relationship between omega-3 PUFAs and esophageal cancer (*p* = 0.83), and the horizontal pleiotropy analysis (*p* = 0.76) generated consistent results. In addition, heterogeneity testing suggested that there was no heterogeneity (*p* = 0.76).

**Figure 2:**
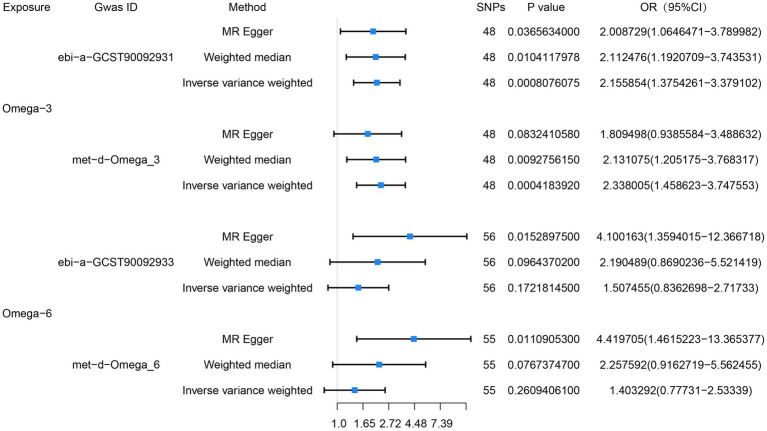
Preliminary MR analysis of the correlation between omega-3, omega-6 and esophageal cancer, and a forest plot of the three MR analysis methods was drawn.

We also obtained data regarding omega-3 and omega-6 PUFA concentrations from another database and performed a second MR analysis as a validation study. For this, we also chose not to use proxy SNPs, and finally included 48 and 55 SNPs, respectively, in the MR analysis. The results of the IVW analysis also showed close relationships of the circulating omega-3 (OR = 2.34, 95% CI = 1.46–3.75, *p* = 4 × 10^−4^) and omega-6 (OR = 1.40, 95% CI = 0.78–2.53, *p* = 0.26) PUFA concentrations with esophageal cancer. MR-PRESSO analysis (*p* = 0.35), and horizontal pleiotropy analysis (*p* = 0.28) indicated the absence of horizontal pleiotropy, and heterogeneity analysis suggesting the absence of heterogeneity. The results of the MR analysis are presented in Figure 2 and the results of the complementary analyses are presented in Table 3.

**Table 3:** Supplementary MR result of omega-3, omega-6, and esophageal carcinoma.

Exposure	Outcome	MR-PRESSO	Cochran’s Q	Pleiotropy_test
Omega-3	esophageal carcinoma	0.840	0.818	0.759
Omega-3^*^	esophageal carcinoma	0.355	0.296	0.278
Omega-6	esophageal carcinoma	0.636	0.648	0.040
Omega-6^*^	esophageal carcinoma	0.711	0.719	0.020
Ratio of omega-3	esophageal carcinoma	0.186	0.077	0.100
Ratio of omega-6	esophageal carcinoma	0.086	0.101	0.182
Omega-3/Omega-6	esophageal carcinoma	0.380	0.274	0.447
Docosahexaenoic	esophageal carcinoma	0.669	0.690	0.089
Linoleic	esophageal carcinoma	0.435	0.479	0.590

### 3.2 Association of the ratios of omega-3 and omega-6 PUFAs to the total fatty acid concentrations with esophageal cancer

**Figure 3:**
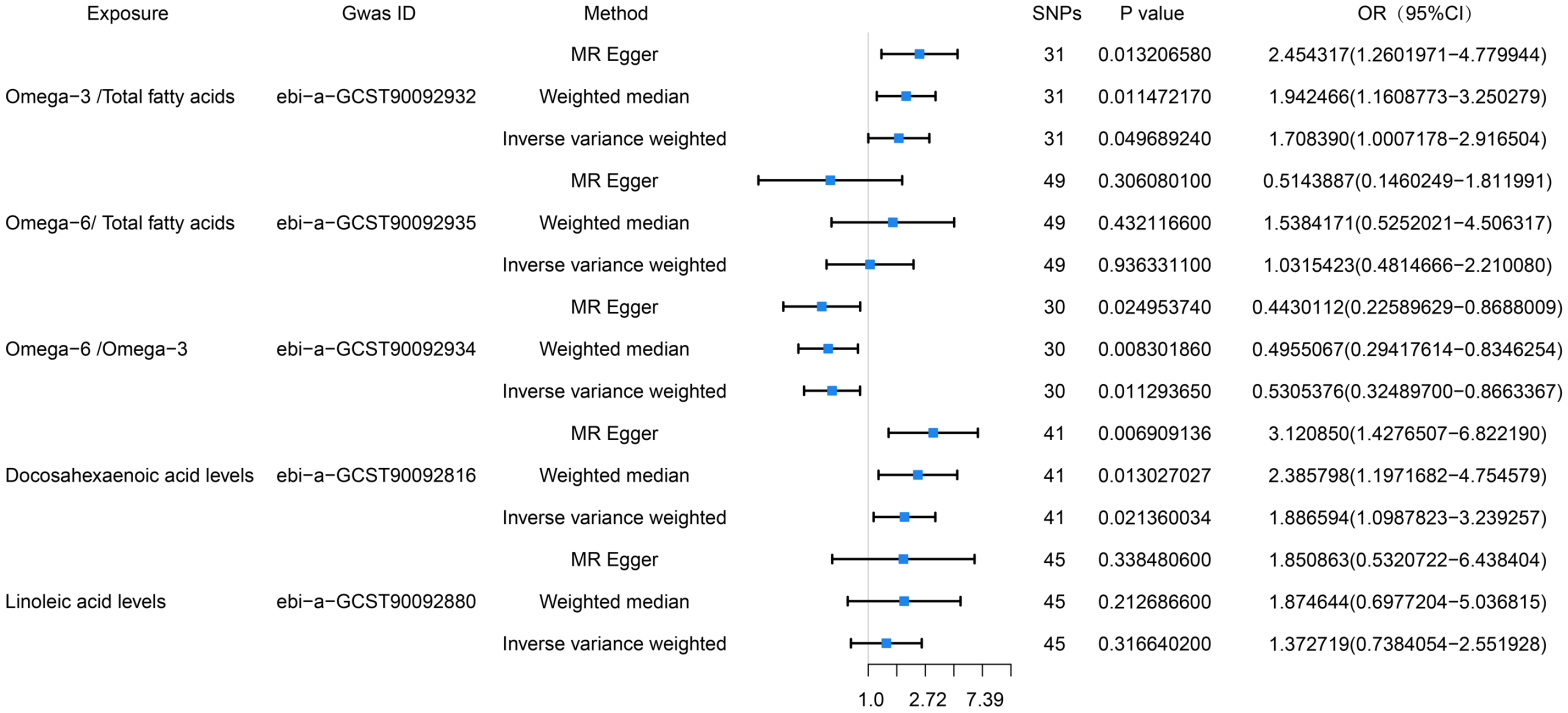
Preliminary MR analysis of the ratio of omega-6 to omega-3, the percentage of both in total fatty acids, docosahexaenoic acid and linoleic acid were plotted as forest plots for the three MR methods in correlation with esophageal cancer.

We also conducted an MR analysis of the relationships of the proportions of omega-3 and omega-6 PUFAs of the total fatty acid concentrations with esophageal cancer, and found that the proportion of omega-3 PUFAs in the circulation positively correlated with the risk of esophageal cancer, whereas there was no significant correlation with respect to the proportion of omega-6 PUFAs. The results of the IVW analyses were as follows: omega-3/total fatty acid ratio: OR = 1.71, 95% CI = 1.00–2.92, *p* = 0.049; omega-6/total fatty acid ratio: OR = 1.51, 95% CI = 0.84–2.72, *p* = 0.17. The results of the MR analysis are presented in Figure 3 and the results of the complementary analyses are presented in Table 3.

### 3.3 Association of DHA and LA with esophageal cancer

Because DHA is the principal omega-3 PUFA, we also performed an MR analysis regarding its relationship with esophageal cancer, and found a positive correlation between its concentration and the risk of esophageal cancer. The results of the IVW analysis were as follows: OR = 1.89, 95% CI = 1.10–3.24, *p* = 0.02. The MR-PRESSO analysis did not detect horizontal pleiotropy (*p* = 0.70), and neither did the horizontal pleiotropy analysis (*p* = 0.08). Furthermore, heterogeneity analysis suggested the absence of heterogeneity (*p* = 0.76). LA is the principal omega-6 PUFA, we also performed an MR analysis regarding its relationship with esophageal cancer, and found no association of its concentration and the risk of esophageal cancer. The results of the IVW analysis were as follows: OR = 1.37, 95% CI = 0.74–2.55, *p* = 0.32. The MR-PRESSO analysis did not detect horizontal pleiotropy (*p* = 0.43), and neither did the horizontal pleiotropy analysis (*p* = 0.59). Furthermore, heterogeneity analysis suggested the absence of heterogeneity (*p* = 0.47). The results of this MR analysis are presented in Figure 3 and the results of the complementary analyses are presented in Table 3.

### 3.4 Association of the circulating concentrations of omega-3 and omega-6 PUFAs with four other esophageal diseases

**Figure 4:**
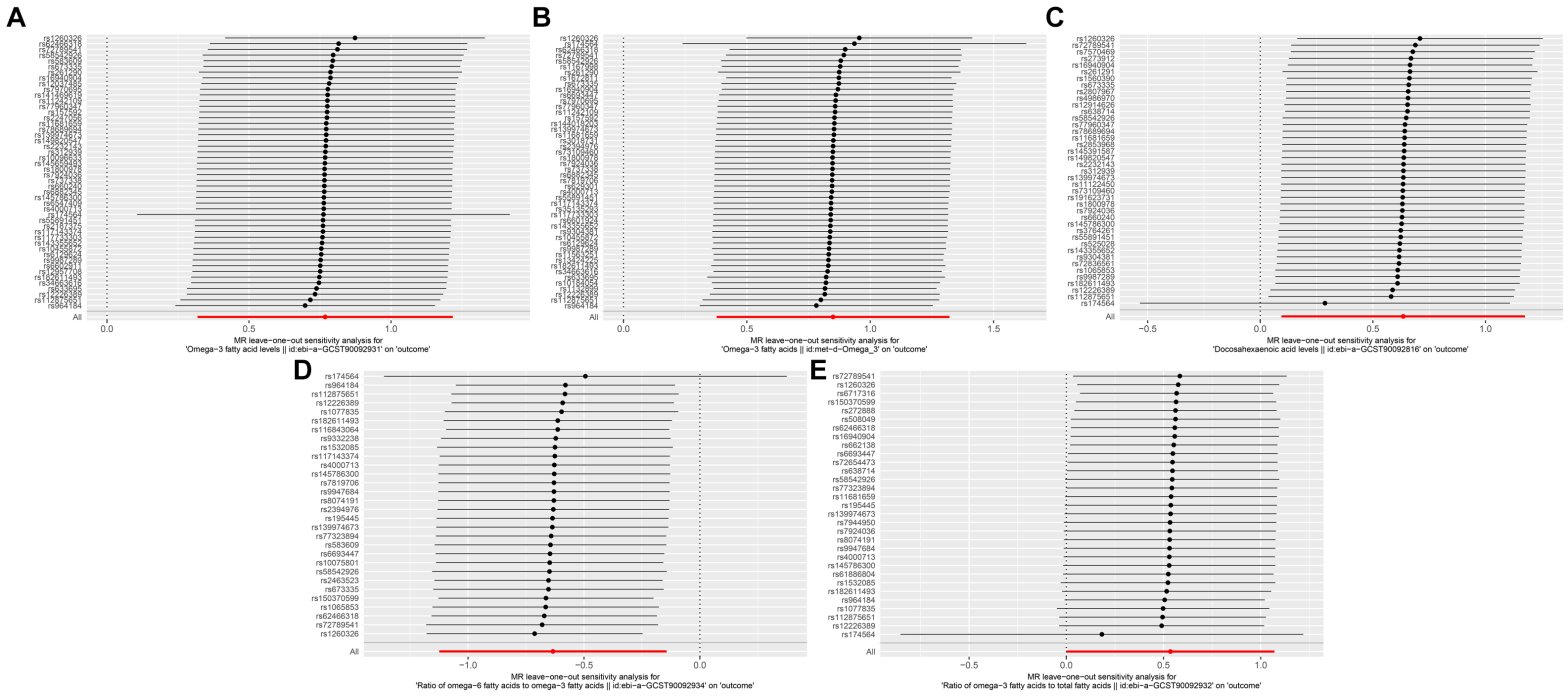
Leave-one-out sensitivity analysis, **(A)** Omega-3 (ebi-a-GCST90092931) and Esophageal Cancer, **(B)** Omega-3 (met-d-Omega 3) and Exophageal Cancer, **(C)** Docosahexaenoic acid and Esophageal Cancer, **(D)** omega-6/omega-3 and Esophageal Cancer, **(E)** omega-3/total fatty acids and Esophageal Cancer.

We found no significant relationships of the circulating concentrations of omega-3 and omega-6 PUFAs with the risk of developing GERD, esophageal ulcer, reflux esophagitis, or Barrett’s esophagus. The result of the IVW analyses for the relationship between omega-3 PUFAs and GERD was: OR = 0.96, 95% CI = 0.91–1.02, *p* = 0.18; for that between omega-3 PUFAs and esophageal ulcer was: OR = 1.00, 95% CI = 0.88–1.14, *p* = 0.988; for that between omega-3 PUFAs and reflux esophagus was: OR = 0.96, 95% CI = 0.88–1.04, *p* = 0.335; for that between omega-3 PUFAs and Barrett’s esophagus was OR = 1.00, 95% CI = 0.93–1.08, *p* = 0.967; for that between omega-6 and GERD was: OR = 0.94, 95% CI = 0.87–1.02, *p* = 0.14; for that between omega-6 and esophageal ulcer was: OR = 0.978, 95% CI = 0.83–1.15, *p* = 0.796; for that between omega-6 and reflux esophagitis was: OR = 0.98, 95% CI = 0.89–1.09, *p* = 0.77; and for that between omega-6 and Barrett’s esophagus was: OR = 0.95, 95% CI = 0.86–1.05, *p* = 0.328. The results of the MR analyses are presented in Table 4 and the results of the supplementary analyses are presented in Table 5. Therefore, leave-one-out sensitivity analyses of positive results consistently indicate that each association is not influenced by individual SNPs. The results of the leave-one-out sensitivity analysis are presented in Figure 4.

**Table 4:** MR result of omega-3, omega-6, and esophageal disease.

Exposure	Outcome	SNPs	Methods	SE	OR (95%CI)	*p* value
omega-3	GERD		MR Egger	0.039	0.9822756 (0.9108767–1.059271)	0.644
		48	Weighted median	0.036	0.9633276 (0.8961703–1.035517)	0.311
			Inverse variance weighted	0.027	0.9641245 (0.9139731–1.017028)	0.180
			MR Egger	0.093	1.0341852 (0.8625414–1.239986)	0.718
omega-3	Ulcer of esophagus	48	Weighted median	0.067	0.9800441 (0.8568837–1.120906)	0.769
			Inverse variance weighted	0.065	1.0009649 (0.8811718–1.137044)	0.988
			MR Egger	0.060	0.9186796 (0.8167725–1.033302)	0.164
omega-3	Reflux esophagitis	48	Weighted median	0.045	0.9185725 (0.8377571–1.007184)	0.071
			Inverse variance weighted	0.042	0.9598754 (0.8831634–1.043251)	0.335
			MR Egger	0.056	1.0299400 (0.9221727–1.150301)	0.603
omega-3	Barrett’s esophagus	48	Weighted median	0.054	1.0242184 (0.9241056–1.135177)	0.648
			Inverse variance weighted	0.040	1.0016081 (0.9262309–1.083119)	0.968
			MR Egger	0.076	0.9784075 (0.8438039–1.134483)	0.774
omega-6	GERD	56	Weighted median	0.054	0.9731946 (0.8742462–1.083342)	0.619
			Inverse variance weighted	0.040	0.9432115 (0.8720341–1.020198)	0.144
			MR Egger	0.158	0.9575270 (0.7021890–1.305714)	0.785
omega-6	Ulcer of esophagus	56	Weighted median	0.109	0.9510280 (0.7623761–1.186362)	0.656
			Inverse variance weighted	0.084	0.9786106 (0.8306678–1.152902)	0.796
			MR Egger	0.100	1.0092409 (0.8299879–1.227207)	0.927
omega-6	Reflux esophagitis	56	Weighted median	0.067	1.0723554 (0.9346917–1.230295)	0.319
			Inverse variance weighted	0.053	0.9847841 (0.8880204–1.092092)	0.771
			MR Egger	0.098	1.0225614 (0.8434589–1.239695)	0.821
omega-6	Barrett’s esophagus	56	Weighted median	0.077	1.0060668 (0.8734432–1.158828)	0.933
			Inverse variance weighted	0.052	0.9500551 (0.8573582–1.052774)	0.328

**Table 5:** Supplementary MR result of omega-3, omega-6, and esophageal disease.

Exposure	Outcome	MR-PRESSO	Cochran’s Q	Pleiotropy_test
omega-3	GERD	0.600	0.550	0.496
omega-3	Ulcer of esophagus	0.112	0.008	0.620
omega-3	Reflux esophagitis	0.032	0.019	0.306
omega-3	Barrett’s esophagus	0.850	0.837	0.487
omega-6	GERD	0.104	0.097	0.569
omega-6	Ulcer of esophagus	0.011	0.009	0.871
omega-6	Reflux esophagitis	0.052	0.057	0.772
omega-6	Barrett’s esophagus	0.639	0.620	0.380

### 3.5 Colocalization analysis of omega-3 and esophageal cancer

We performed a colocalization analysis of omega-3 PUFAs and esophageal cancer. A positive result is defined as H4 > 0.8; however, our analysis showed that H4 was consistently <0.8, indicating no evidence of colocalization. The analysis results are presented in Supplementary Table S1.

## 4 Discussion

To the best of our knowledge, this is the first MR study of the relationships of omega-3 and omega-6 PUFAs with esophageal cancer and esophageal diseases. Furthermore, we performed a subanalysis of the relationship between DHA, the principal omega-3 PUFA in food, and esophageal cancer. We found a positive correlation between circulating omega-3 PUFA concentration and esophageal cancer, but no association with other esophageal diseases, and there was no association of the circulating omega-6 PUFA concentration with esophageal cancer or other esophageal diseases, and there was no association of the circulating LA concentration with esophageal cancer there was a positive association between the circulating DHA concentration and esophageal cancer. However, we found a negative association of the circulating omega-6/omega-3 concentration with esophageal cancer. In summary, our study suggests that circulating omega-3 and DHA concentrations may be risk factors for the development of esophageal cancer, whereas an increased omega-6/omega-3 ratio may serve as a protective factor against the incidence of esophageal cancer.

### 4.1 Status of omega-3 PUFA research in other diseases

Research to date has principally been focused on the relationships of omega-3 PUFAs with chronic diseases such as cardiovascular disease and cancer, and this has primarily involved investigation of the roles of their antioxidant and anti-inflammatory effects in disease onset and progression. It has been suggested that omega-3 PUFAs may be beneficial for patients at risk of a number of diseases and may represent means of both preventing and treating these diseases (18). A previous meta-analysis showed that supplementation with omega-3 PUFAs alone reduces the risk of cardiovascular events in patients with diabetes, possibly because they modulate the production of a number of anti-inflammatory substances that can promote tissue repair and ameliorate inflammation during atherosclerosis (19, 20). This finding was validated in a pooled and harmonized analysis of 29 prospective studies, which showed that supplementation with omega-3 PUFAs is associated with a lower risk of ischemic stroke, probably because omega-3 PUFAs reduce platelet count, reduce arterial stiffness, and improve endothelial function (21). Although findings regarding the anti-inflammatory effects of omega-3 PUFAs have been inconsistent, a large number of studies have shown that they protect against the development of such disease. However, PUFAs exhibit a wide range of bioactivities at both the molecular and cellular levels, and therefore we should exercise caution regarding their use as nutritional supplements.

### 4.2 Status of omega-3 PUFA research in tumor

Our research findings demonstrate a positive correlation between circulating omega-3 PUFAs concentrations and the incidence of esophageal cancer, suggesting that elevated circulating omega-3 PUFAs concentrations may be a risk factor for the development of esophageal cancer. In another meta-analysis of data from 67 prospective studies involving 310,955 participants, high omega-3 PUFA concentrations were found to be associated with a lower risk of colorectal cancer (6). Thus, omega-3 PUFAs may represent a risk factor for, or a means of preventing the development of, malignancy. In one prospective study, the consumption of omega-3 PUFAs in the diet or as supplements was found to increase the risk of endometrial cancer in women with overweight or obesity (11), and a recent meta-analysis generated consistent findings (22). Our findings align with similar results obtained in other studies, all of which conclude that omega-3 may act as a potential risk factor for tumor development. Furthermore, a meta-analysis of data from 47 randomized controlled trials showed that increasing long-chain omega-3 PUFA consumption may have little effect on the risk of a diagnosis of cancer or cancer-related mortality, but may slightly increase the risk of prostate cancer (10). Thus, the inconsistency of previous findings regarding the relationship between omega-3 PUFAs and malignancy are evident and may be related to their many biological activities. However, some prior studies have indicated that omega-3 PUFAs may possess anti-cancer properties or enhance the efficacy of chemotherapy in tumor treatment, serving as a nutritional supplement for disease prevention (23). A review study discussed the role of omega-3 PUFAs in tumor complications, highlighting their anti-inflammatory and protective effects due to their involvement in the resolution of inflammation. The findings suggested that omega-3 PUFAs and their metabolites might regulate key pathways in cancer-related complications (24). Furthermore, another review analysis on breast cancer indicated that omega-3 PUFAs supplementation could serve as an adjunct to chemotherapy or other conventional anti-tumor treatments (25). This may represent a future research direction for polyunsaturated fatty acids such as omega-3 PUFAs. In summary, the current research on omega-3 PUFAs is insufficient, thus warranting further discussion regarding their use as nutritional supplements.

### 4.3 Status of research on docosahexaenoic acid

The principal omega-3 FAs in food are DHA and EPA, and these have been shown to have similar anti-inflammatory and antioxidant effects to omega-3 PUFAs as a whole in previous studies. In a randomized controlled study, DHA was found to have a superior effSect to EPA on specific markers of inflammation and circulating lipid concentrations (26). Specifically, DHA caused a larger reduction in the circulating concentrations of IL-8 and triglycerides. However, the anti-inflammatory effects of DHA are not necessarily beneficial, and in a recent secondary analysis of a prospective cohort study, DHA was found to be a potential risk factor for poor appetite during chemotherapy for early breast cancer (27). These contrasting findings show that future studies of omega-3 PUFAs should be more detailed, and that subgroup studies of specific PUFAs should also be performed.

### 4.4 Status of research on omega-6 PUFAs

There have also been contradictory findings regarding the roles of omega-6 PUFAs in disease. In a systematic evaluation of data from 19 randomized controlled trials, it was shown that omega-6 PUFAs may have no or little effect on all-cause mortality or cardiovascular events, but that they may reduce the risk of myocardial infarction. However, because of the low quality of the evidence, there is much uncertainty regarding the relationships of omega-6 PUFAs with all-cause mortality and cardiovascular events (28). There is also uncertainty regarding the relationship between omega-6 PUFAs and the development of malignancy. In one prospective study, they were shown to be positively associated with the development of ER + PR+ breast cancer (29), but in a meta-analysis, no significant association with cancer risk was identified (30). Thus, there is a great deal of controversy regarding the relationship between omega-6 PUFAs and the progression of disease, and more high-quality evidence is needed to better evaluate this.

### 4.5 Status of *in vitro* testing and omega-6/omega-3 research

It is not easy to perform studies regarding the effects of dietary components on human health; therefore researchers have conducted a large number of *in vitro* and animal-based studies. In one *in vitro* study, omega-3 and omega-6 PUFAs were found to increase or inhibit the metastatic potential of gastric cancer via COX-1/PGE3 and COX-2/PGE2, respectively (31). In an animal study, it was shown that the activation of TLR4 in rats is inhibited by a high dietary omega-6/omega-3 PUFA ratio, which in turn reduces their circulating lipid concentrations, improves their glucose tolerance, and ameliorates their insulin resistance (32). Our research findings indicate a negative correlation between the ratio of circulating omega-6/omega-3 concentrations and the incidence of esophageal cancer. This suggests that an increased ratio of circulating omega-6/omega-3 concentrations is associated with a reduced risk of developing esophageal cancer. These results are consistent with the conclusions of many previous studies. In a clinical study, the omega-6/omega-3 PUFA ratio was also shown to be associated with health, and in a meta-analysis, it was shown that a high omega-6/omega-3 PUFA ratio reduces the risk of breast cancer (33). Finally, in another study, it was shown that an appropriate omega-6/omega-3 ratio may help control obesity, as well as playing a key role in disease prevention (34). Our findings are consistent with those of similar studies. Several studies on omega-6/omega-3 have elucidated that different ratios might yield opposite effects on various diseases, potentially serving a preventive or therapeutic role, but also possibly contributing to disease progression (35, 36). Our study employed Mendelian randomization, which is a qualitative research method, and the results indicate only an association, precluding quantitative analysis. Therefore, we currently cannot determine the optimal ratio for the prevention of esophageal cancer. Future research should focus on quantitative studies of omega-6/omega-3 ratios.

### 4.6 Limitation

There were several limitations to the present analysis. First, most of the data we used were GWAS data relating to European populations, and therefore genetic diversity analyses of other populations are needed to generalize the conclusions. Second, although we used several methods and took rigorous steps to avoid horizontal pleiotropy, genetic variation is extremely complex and we were unable to completely eliminate horizontal pleiotropy. Therefore, studies with larger sample sizes and more advanced methods are required to further validate the results. Finally, because of the limitations of the databases, we did not evaluate the individual relationships of each of the omega-3 PUFAs with esophageal disease.

## 5 Conclusion

We found that elevated circulating concentrations of omega-3 PUFAs might be a risk factor for the development of esophageal cancer. Conversely, a higher omega-6/omega-3 ratio might serve as a protective factor against esophageal cancer. Currently, the widespread use of omega-3 PUFAs as nutritional supplements warrants further evaluation, and the underlying mechanisms require additional investigation.

## Data availability statement

The original contributions presented in the study are included in the article/Supplementary material, further inquiries can be directed to the corresponding author.

## Ethics statement

Ethical approval was not required for the study involving humans in accordance with the local legislation and institutional requirements. Written informed consent to participate in this study was not required from the participants or the participants’ legal guardians/next of kin in accordance with the national legislation and the institutional requirements.

## Author contributions

WC: Conceptualization, Data curation, Investigation, Methodology, Software, Validation, Visualization, Writing – original draft, Writing – review & editing. MC: Conceptualization, Data curation, Investigation, Methodology, Software, Validation, Visualization, Writing – original draft, Writing – review & editing. JH: Conceptualization, Investigation, Software, Validation, Visualization, Writing – original draft, Writing – review & editing. QX: Data curation, Investigation, Validation, Writing – original draft, Writing – review & editing. YH: Data curation, Investigation, Validation, Writing – original draft, Writing – review & editing. CC: Formal analysis, Funding acquisition, Project administration, Resources, Writing – original draft, Writing – review & editing. YZ: Formal analysis, Funding acquisition, Project administration, Resources, Writing – original draft, Writing – review & editing.

## Footnotes

^1^https://gwas.mrcieu.ac.uk/

^2^https://www.ebi.ac.uk/
