# Supplementary material for: Relationships of omega-3 and omega-6 polyunsaturated fatty acids with esophageal diseases: a two-sample Mendelian randomization analysis
Source: Front Nutr. 2024 Jul 17;11:1408647. doi: 10.3389/fnut.2024.1408647 (PMC11288942; doi:10.3389/fnut.2024.1408647)
Supplement: Supplementary file 1 [file Table_1.docx]

| **TableS1.**Supplementary Data on F-Statistics and Colocalization Analysis of SNPs with Partial Positive Results | | | | | | | | | |
| --- | --- | --- | --- | --- | --- | --- | --- | --- | --- |
| **SNP** | **POS** | **EA** | **OA** | **Beta** | **EAF** | **SE** | **P value** | **F-statistic** | **coloc pp.H4** |
| rs10096633 | 19830921 | T | C | -0.0418412 | 0.123902 | 0.00599603 | 3.00E-12 | 48.69373818 | 1.90E-07 |
| rs10455872 | 161010118 | G | A | -0.0617415 | 0.078987 | 0.00733401 | 3.80E-17 | 70.87022386 | 7.61E-07 |
| rs11242109 | 131677047 | T | G | 0.0234291 | 0.479035 | 0.00395796 | 3.20E-09 | 35.03973861 | 1.66E-07 |
| rs112875651 | 126506694 | A | G | -0.0874051 | 0.392337 | 0.00410836 | 1.90E-100 | 452.6150713 | 4.21E-07 |
| rs11681659 | 136820960 | T | C | -0.0264162 | 0.716465 | 0.00436662 | 1.50E-09 | 36.59673458 | 6.08E-08 |
| rs117143374 | 40555561 | C | T | -0.03454 | 0.14225 | 0.00569242 | 1.30E-09 | 36.81656296 | 4.86E-07 |
| rs117733303 | 160922870 | G | A | -0.110595 | 0.018512 | 0.0146728 | 4.80E-14 | 56.81165455 | 1.64E-06 |
| rs12037485 | 2332391 | T | C | 0.0222781 | 0.460471 | 0.00396786 | 2.00E-08 | 31.52361863 | 3.18E-07 |
| rs12226389 | 61823630 | C | T | -0.0535751 | 0.185805 | 0.00510749 | 9.70E-26 | 110.0280404 | 5.62E-07 |
| rs1260326 | 27730940 | C | T | -0.0830756 | 0.604008 | 0.00404264 | 7.70E-94 | 422.2885275 | 4.16E-07 |
| rs12957708 | 47284353 | G | A | 0.0311923 | 0.14228 | 0.00568503 | 4.10E-08 | 30.10382801 | 6.21E-07 |
| rs139974673 | 44027885 | C | T | 0.118563 | 0.025916 | 0.0124678 | 1.90E-21 | 90.42971307 | 3.52E-07 |
| rs141469619 | 116714293 | G | A | 0.117162 | 0.010139 | 0.0208113 | 1.80E-08 | 31.69330873 | 2.21E-06 |
| rs143355652 | 61453822 | T | C | -0.153201 | 0.010466 | 0.0198593 | 1.20E-14 | 59.50970189 | 2.20E-06 |
| rs145659493 | 61850279 | A | C | 0.107728 | 0.015853 | 0.0158266 | 1.00E-11 | 46.33128966 | 1.30E-06 |
| rs145786300 | 61406089 | A | G | -0.150855 | 0.011993 | 0.0187118 | 7.50E-16 | 64.99511731 | 1.97E-06 |
| rs149820547 | 61983775 | G | T | -0.0678421 | 0.042105 | 0.00988147 | 6.60E-12 | 47.13547629 | 1.52E-08 |
| rs157592 | 45424514 | C | A | 0.0325574 | 0.185273 | 0.00522115 | 4.50E-10 | 38.88297958 | 2.19E-07 |
| rs16940904 | 44186063 | T | C | -0.0345078 | 0.226566 | 0.00474779 | 3.60E-13 | 52.82554535 | 4.13E-07 |
| rs174564 | 61588305 | G | A | -0.336931 | 0.347009 | 0.00412782 | 1.00E-200 | 6662.433502 | 4.59E-07 |
| rs1800978 | 107665978 | G | C | -0.0387593 | 0.123988 | 0.00603019 | 1.30E-10 | 41.31257879 | 2.92E-07 |
| rs182611493 | 19458388 | G | A | -0.220029 | 0.012519 | 0.0190586 | 7.80E-31 | 133.2816335 | 2.03E-06 |
| rs2187375 | 47172283 | G | A | 0.0524746 | 0.822879 | 0.00518371 | 4.40E-24 | 102.4729707 | 3.71E-07 |
| rs2232143 | 60899701 | C | T | 0.104052 | 0.021879 | 0.0141105 | 1.70E-13 | 54.37615636 | 3.24E-07 |
| rs2247056 | 31265490 | C | T | 0.0356403 | 0.703307 | 0.00432842 | 1.80E-16 | 67.7979183 | 5.13E-08 |
| rs2288912 | 45449199 | G | C | 0.026507 | 0.496385 | 0.00396428 | 2.30E-11 | 44.70797101 | 4.25E-07 |
| rs261290 | 58678720 | C | T | -0.112905 | 0.654648 | 0.00416818 | 1.40E-161 | 733.7124133 | 1.97E-07 |
| rs312939 | 21409548 | A | G | 0.034251 | 0.752467 | 0.00458283 | 7.80E-14 | 55.85620998 | 8.88E-08 |
| rs34663616 | 58569330 | A | C | 0.035253 | 0.137651 | 0.00586293 | 1.80E-09 | 36.15390324 | 6.45E-07 |
| rs4000713 | 25990597 | A | G | -0.028225 | 0.295416 | 0.0043421 | 8.00E-11 | 42.25330711 | 2.38E-07 |
| rs55891451 | 96728169 | C | A | 0.033166 | 0.20172 | 0.00494622 | 2.00E-11 | 44.96056724 | 3.69E-07 |
| rs583609 | 62916796 | C | T | -0.0711993 | 0.352508 | 0.00414084 | 2.90E-66 | 295.642588 | 9.06E-08 |
| rs58542926 | 19379549 | T | C | -0.172929 | 0.074379 | 0.00754654 | 3.30E-116 | 525.0883114 | 1.75E-07 |
| rs6129624 | 39167592 | A | G | -0.0242913 | 0.335232 | 0.0042636 | 1.20E-08 | 32.45944243 | 4.02E-07 |
| rs62466318 | 73042085 | T | C | -0.0716979 | 0.204201 | 0.00492892 | 6.20E-48 | 211.5932357 | 4.59E-07 |
| rs633695 | 58725839 | G | A | 0.0848151 | 0.292334 | 0.00436378 | 3.80E-84 | 377.7575119 | 4.14E-07 |
| rs6547409 | 21190209 | T | C | -0.0565011 | 0.051259 | 0.0090697 | 4.70E-10 | 38.80792384 | 8.04E-07 |
| rs660240 | 109817838 | C | T | 0.0370395 | 0.784707 | 0.00481655 | 1.50E-14 | 59.13580761 | 2.23E-07 |
| rs6602911 | 114547372 | T | C | 0.0231802 | 0.360074 | 0.00412043 | 1.80E-08 | 31.64766872 | 4.19E-07 |
| rs673335 | 75450576 | C | T | -0.0684035 | 0.159752 | 0.00539262 | 7.20E-37 | 160.8975603 | 3.25E-07 |
| rs6882345 | 156397673 | A | G | 0.0274277 | 0.63285 | 0.00409997 | 2.20E-11 | 44.75173431 | 1.70E-07 |
| rs72789541 | 15127534 | A | T | -0.0834033 | 0.295973 | 0.00434225 | 3.20E-82 | 368.9175102 | 2.12E-07 |
| rs737338 | 11347657 | T | C | -0.0674466 | 0.035184 | 0.0107428 | 3.40E-10 | 39.41645747 | 8.66E-07 |
| rs77960347 | 47109955 | G | A | 0.157178 | 0.013234 | 0.0172904 | 9.90E-20 | 82.63535233 | 8.22E-07 |
| rs78689694 | 126234820 | C | G | 0.0326453 | 0.133362 | 0.00581875 | 2.00E-08 | 31.47562618 | 1.25E-07 |
| rs7924036 | 65191645 | T | G | 0.0222276 | 0.504218 | 0.0039569 | 1.90E-08 | 31.55494615 | 1.34E-07 |
| rs7970695 | 121423376 | A | G | -0.0235143 | 0.620564 | 0.0040847 | 8.60E-09 | 33.13875789 | 2.28E-07 |
| rs964184 | 116648917 | C | G | -0.117565 | 0.867233 | 0.005805 | 3.40E-91 | 410.151303 | 6.60E-07 |
| rs9987289 | 9183358 | G | A | 0.0544031 | 0.909161 | 0.00688493 | 2.70E-15 | 62.43680682 | 7.33E-07 |
